# Neuralgia—Sulphate of Nickel

**Published:** 1870-01

**Authors:** 


Neuralgia.—Sulphate of Nickel.—A case of obstinate neuralgia is related which was cured by sulphate of nickel, in doses of half a grain, three times a day. At the end of a week a dose of one grain was given. Its sedative action was speedily manifested in reducing the pulse and procuring sleep. All symptoms of the paroxysm disappeared. The remedy seems worth a trial.—*Physician and Pharmaceutist.*

